# Global attitudes on and the status of enteral nutrition therapy for pediatric inflammatory bowel disease

**DOI:** 10.3389/fmed.2022.1036793

**Published:** 2022-12-08

**Authors:** Juan Luo, Yong-Mei Xie, Mei Wu, Jin-Gui Zhao, Liang-Liang Hu

**Affiliations:** ^1^Department of Pediatrics, West China Second University Hospital, Sichuan University, Chengdu, Sichuan, China; ^2^Key Laboratory of Birth Defects and Related Diseases of Women and Children, Sichuan University, Ministry of Education, Chengdu, Sichuan, China; ^3^Department of Pediatrics, Luzhou People’s Hospital, Luzhou, Sichuan, China

**Keywords:** inflammatory bowel disease, enteral nutrition, children, implementation status, questionnaire

## Abstract

Enteral nutrition (EN) is a diet-remission therapy for inflammatory bowel disease (IBD) that plays a more important role in children than adults. EN includes exclusive enteral nutrition (EEN), partial enteral nutrition (PEN), and maintenance enteral nutrition (MEN). However, EEN remains an unstandardized treatment for pediatric IBD. The types and methods of EN differ around the world. The current study reviewed the EN literature on children with IBD. A total of 12 survey studies were identified that analyzed the current state of EN use, including clinical opinions, implementation methods, treatment course, EEN formula, IBD classification, progress, dietary reintroduction, and patient feedback. The findings revealed that EEN has a strong effect on mild to moderate Crohn’s disease (CD). The usage rates of this treatment in different sites were ileum/colon (Paris classification L3) > ileum (L1) > upper digestive tract (L4) > colon (L2) > perianal disease (P) > ulcerative colitis (UC) > extraintestinal lesions. The polymeric formula was the most used EN formulation. New EN diets include a CD exclusion diet (CDED), a specific carbohydrate diet (SCD), and a CD treatment-with-eating (CD-TREAT) diet. Children with IBD responded similarly to EEN administered orally or using a feeding tube. Most guidelines recommended 6–8 weeks of EEN treatment to induce remission. Many clinicians preferred to combine drug medications during EEN and recommended that MEN accounts for at least 25–35% of daily caloric intake. EN remains an unstandardized therapy that requires teamwork across disciplines.

## Introduction

Inflammatory bowel disease (IBD) includes Crohn’s disease (CD), ulcerative colitis (UC), and unclassified inflammatory bowel disease (IBD-U). Enteral nutrition (EN), which includes exclusive enteral nutrition (EEN), partial enteral nutrition (PEN), and maintenance enteral nutrition (MEN), is a food-induced therapy for IBD remission. Due to its safety profile, this treatment is commonly used in children. EEN is recommended as the first line of treatment for CD remission, especially among children with active luminal CD (1). This review summarizes the results of 12 survey studies and provides an update on the global status of EN use for pediatric IBD to standardize the treatment.

## Literature search and screening

**FIGURE 1:**
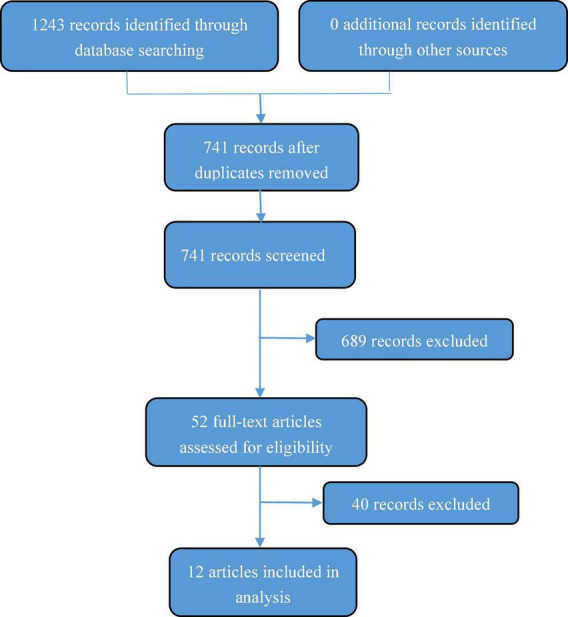
Study flow diagram.

PubMed, Embase, Cochrane Library, CNKI, and CBM databases were searched for global studies on EN treatment of pediatric IBD. Studies published from the establishment of each database to December 2021 were included in the search. A combination of subject headings and free words, including inflammatory bowel disease, Crohn’s disease, ulcerative colitis, enteral nutrition, and children, were used as the search terms. Studies were included if the subjects were children with IBD who were ≤18 years of age, the intervention included EN, the outcome measures included patient attitudes and implementation of EEN among pediatric patients with IBD, and the study was survey-based. Studies were excluded if they were duplicate reports, articles from which the original text could not be obtained, or articles lacking the required information. Two researchers independently screened the literature and cross-checked the data. Disagreements were resolved by discussion or consultation with a third party. Literature screening was performed by first reading the title and excluding irrelevant literature. Further reading of the abstract and full text was then performed to determine whether the study should be included. If necessary, the original authors were contacted by mail or telephone to obtain unidentified information. Several variables were extracted, including research title, first author, publication journal, publication period, survey country or region, and key results. A total of 1,243 relevant studies were obtained during the initial inspection. After a layer-by-layer screening, 12 survey studies were considered highly relevant and selected for further analysis (Study flow diagram in Figure 1). These studies reflected the implementation status of pediatric EN, including regional variation, time evolution, clinical opinions, and patient attitudes. Analysis results of the 12 studies (ART12Q) are summarized in Table 1.

**TABLE 1:** Global questionnaire survey on exclusive enteral nutrition (EEN) implementation of inflammatory bowel disease (IBD) children.

Refere- nces	Infor- mant	Investi- gated area	EEN usage rate	Treatment goal	Disease location	Severity of illness	EEN formula	Dietary supplemen- tation during EEN	Feeding pattern	Treatment course	MEN	Food reintro- duction	Drug use during EEN	Factors affecting EEN implemen- tation
(49)	Pediatric gastroen- terology center	Australia/New Zealand	No data	No data	No data	No data	Polymeric formula (94%), elemental formula (6.3%)	Flavoring is allowed (44%), small amounts of extra food and drinks (sweets and liquids) are allowed (56%), and only water is allowed (44%)	NG (16%)	6 W (11%), 6–8 W (50%), 8 W (39%)	Usage rates (88%), the recomm- ended calories are about 24% of total intake	<1 W (17%), 1–4 W gradual introduc- tion (78%), temporary low fiber/low residue diet (78%)	No data	Medical team (100%), family (100%), patient compliance (83.3%), economic cost (72.2%), formulation type (50%), disease severity (55.6%)
(16)	Patient/Guardian	USA (Children’s Hospital Colorado)	46.1%	Induced remission (40%), sustained remission (16%), uncertain (2%)	No data	Mild to moderate (68%), severe (32%)	No data	No data	NG (24.5%), colostomy mouth (2%)	<2 W (8%), 2–4 W (4%), 4–6 W (15%), 6–8 W (4%), 8–12 W (20%), >12 W (49%)	No data	No data	No data	Economic cost (33%), social difficulty (27%), formula type (23%), difficulty of tube feeding (18%)
(15)	PGE	Australia/New Zealand	84%	Any time (98%), induced remission (new diagnosis 100%, recurrence 86%)	CD 100% (L2 72%, L3 86%, L4 73%, P 25%) UC 8%, IBD-U 30%	Mild (new diagnosis 73%, recurrence 62%) Moderate (new diagnosis 89%, recurrence 89%) Severe (new diagnosis 87%, recurrence 76%)	Polymeric formula (75%), Semi elemental formula (5.4%), elemental formula (8.1%)	Flavoring is allowed (48.6%) and other liquids besides water are allowed (27%)	No data	6 w (5%), 6–8 w (95%)	usage rates (51%), the recomm- ended calories are about 30–50% of total intake	Gradual reintro- duction (76%), low residue diet first (45%), low allergen diet first (17%)	CS (3%), 5-ASA (16%), AZA (68%), MTX (32%)	Medical team (97%), family (100%), patient compliance (97%), disease site (68%), formulation type (65%), economic cost (46%), disease severity (43%)
(17)	Common PGE (65%), PGEIBD (21%), dietician (10%)	26 countries	63%	Induced remission (new diagnosis 82%, recurrence 38%)	L1 88%, L2 52%, L3 91%, *P* < 31, UC < 6%	No data	Polymeric formula (88%)	No intake of any food other than water (63%), 31% of common PGE and 26% of PGE-IBD allow intake of small amounts of other foods (candies and liquids)	Po (66%) NG (33%)	4–6 w (2%), 6 w (31%), 8 w (57%), 8–12 W (7%)	No data	No data	No data	Medical team (21%), economic cost (19%), formula type (58%)
(18)	Pediatric CD therapist, patient/guardian	Japan	Doctor 84%, patient 70%	Induced remission (82% new diagnosis, 59.1% recurrence)	L1 (16%), L2 (17%), L3 (48%)	No data	Elemental formula (doctor 85%, patient 98%)	No data	No data	An average of 15.9 d	Usage rates (63.7%) The recom- mended calories are about 30% of total intake	No data	CS (40%), immuno- modulator (36%), 5-ASA (97%), biologics (21%), ABX (12%)	Medical staff and family support are major factors
(51)	Patient/Guardian	UK	No data	Induced remission (new diagnosis 76%, relapse 24%)	No data	No data	No data	No data	Po (45%) NG (55%)	8 W (79%)	No data	No data	No data	No data
(52)	Pediatric gastroen- terology unit	Spain	90%	Induced remission (new diagnosis 70.6%, recurrence 83.3%, nutritional development 96.1%)	Any part of the digestive tract (62.7%), L1 (37%), L3 (37%), L4 (69%), the intestinal outside (50%)	Mild to moderate (100%)	Polymeric formula (70.6%)	Flavoring was allowed (60.8%), only water was allowed (90.2%), and other foods were allowed (9.3%)	Po is preferred	6 W (19.6%) 6–8 W (22.5%), 8 W (47.1%)	Usage rates (88.4%)	Gradual reintro- duction over a variable period of time	CS (20%), immuno- modulator (95%), 5-ASA (65%), ABX (69%)	Family (71%), patient compliance (71%), healthcare team (69%), formulation type (30%), economic cost (10%), difficulty in tube feeding (8%)
(53)	Pediatric center	Europe, North America and Asia Pacific countries	89%	Induced remission (new diagnosis 94%, recurrence 97%)	No data	No data	Polymeric formula (90%), Semi elemental formula (32%), Elemental formula (48%)	Flavorings are allowed (81%), most allow water and no other liquids (16%)	Po (56%), NG (37%), colostomy mouth (7%)	4–6 W (3.2%), 6–8 W (81%), 812 w (16.1%)	Usage rates (87%)	The time is 1–12 W. Gradual reintro- duction (52%), initial low-fiber diet (26%), specific food recomme- ndations (39%)	5-ASA (100%), CS (50%), AZA (50%), ABX 50%	No data
(54)	PGE	USA, Canada, Mexico	83%	Induced remission (83%)	CD 83% (L3 79%, L4 76% (P 20%), UC 33%	No data	Polymeric formula (47%), semi elemental formula (55%), elemental formula (47%)	No data	NG (71%)	6 w (30%), 6–8 w (46%), 8 w (25%), 8–12 w (25%)	Use it often or always (7%)	Go straight back to the regular diet (27%) and gradually reintro- duce (57%), low fiber/residue first (55%), low allergen first (32%)	Overall drug use (USA 63%, Canada 24%), 5-ASA (69%), CS (51%), 6-MP (60%), AZA (40%), infliximab (40%), MTX (12.6%)	Patient compliance (72%)
(55)	Pediatric unit of IBD	Sweden	96%	Induced remission (new diagnosis 65%, recurrence 25%)	CD (96%) UC (4%)	Mild to moderate (96%)	Polymeric formulas (54%)	Allow some accompanying food (candy and liquid) (81%)	Po 39%, NG (61%)	4–6 W (12%), 6 W (52%), 6–8 W (32%)	Usage rates (100%)	No data	CS (69%), immuno- modulator (76%), 5-ASA (79%), antiTNF (21%), ABX (62%)	Compliance, discomfort with tube feeding, and psychological and social difficulties
(56)	PGE	Australia	57%	Induced response (100%), maintained response (76%), nutritional development (81%)	CD 100% (L3 75%, L4 67%), UC 19%	No data	Polymeric formulas (92%)	No data	Po (66.7%), NG (33.3%)	6–8 W (92%)	Usage rates (50%)	Gradual reintro- duction (66.7%)	Most doctors do not use EEN in combi- nation with drugs	Compliance of children (95.2%), family (61.9%), medical team (47.6%), cost (28.6%)
(57)	Pediatric gastroen- terologist	16 countries	24.6% (USA 4.3%, Canada 36%, Western Europe 61.8%, Israel 19.2%)	Induced response (100%)	No data	Mild to moderate (59.9%), severe (14.3%)	Polymeric formulation 39% (USA 18%, Canada 20%, Western Europe 79%, Israel 73%)	No data	No data	No data	No data	No data	No data	No data

IBD, inflammatory bowel disease; CD, Crohn’s disease; UC, ulcerative colitis; IBD-U, unclassified inflammatory bowel disease; EEN, exclusive enteral nutrition; PEN, partial enteral nutrition; MEN, maintenance enteral nutrition; CS, corticosteroids; 5-ASA, 5-aminosalicylic acid; anti-TNF, anti-tumor necrosis factor; AZA, azathioprine, 6-MP, 6-mercaptopurine; MTX, methotrexate; ABX, antibiotics; NG, nasogastric tube; PCDAI, pediatric Crohn’s disease activity index; ESR, erythrocyte sedimentation rate; PLT, platelet count; PGE, pediatric gastroenterologist. Disease location (Paris classification): L1, distal 1/3 ileum, with or without cecal lesions; L2, colonic lesions; L3, ileocolonic lesions; L4, lesions of the upper digestive tract; P, perianal lesions.

## Exclusive enteral nutrition treatment of different inflammatory bowel disease types and lesion sites

Exclusive enteral nutrition is effective at inducing the remission of intraluminal CD (1); however, only a few studies recommend the use of this treatment for active perianal lesions and pediatric UC (2), and the supporting data are insufficient. There is also little evidence to support the use of EEN for isolated extra-gastrointestinal lesions and isolated oral lesions (3). ART12Q found that the use of EEN differed by lesion site, with ileal/colonic lesions > ileal lesions > upper gastrointestinal lesions > colonic lesions > perianal lesions. However, EEN-induced remission is not significantly associated with the lesion site (1), thus it is not necessary to consider this variable in the treatment of children with CD.

## Exclusive enteral nutrition treatment for “induction remission/maintenance remission” and “new onset/recurrence”

**FIGURE 2:**
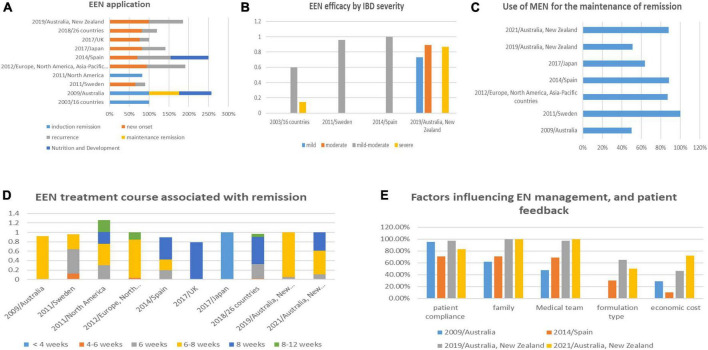
Comparison of the application of EN in pediatric IBD in various questionnaires.

Exclusive enteral nutrition is primarily used to induce IBD remission. Some meta-analyses and prospective studies have shown that EEN is as effective as corticosteroids (4, 5) and biologics (infliximab) (6) at promoting pediatric CD remission (7–10), and more effective than corticosteroids at inducing mucosal healing (11, 12). Frivolt et al. reported a 92% response rate after the first course of EEN therapy. In several retrospective studies, the rate of remission was 58.3–80% after the second course (13). ART12Q found that most clinicians agreed that EEN was effective at inducing the remission of new-onset disease and used this treatment to induce the remission of recurrent cases in some areas (Figure 2A). Indeed, the European Society of Pediatric Gastroenterology, Hepatology, and Nutrition (ESPGHAN) (14) concluded that EEN treatment can be revisited in cases of recurrence. While EEN is effective at maintaining remission (3), ART12Q found that it was not widely adopted by doctors (Figure 2A) and that patient compliance was extremely low. A total of 11 studies reported the rate of recurrence after EEN-induced remission (14), and these values ranged from 2 to 67% within 1 year and 58–68% within 2 years with a median recurrence time of 6.5–12.7 months. Thus, EEN is recommended as the first-line therapy for inducing remission of newly diagnosed CD in children and is suggested for use in treating recurrent cases and maintaining remission as needed.

## Exclusive enteral nutrition efficacy by inflammatory bowel disease severity

Analysis results of the 12 studies found that most pediatricians approved the efficacy of EEN to treat mild-moderate IBD, but showed that EEN efficacy against severe CD was relatively low (Figure 2B). ESPGHAN (14) identified that EEN could promote the mucosal healing of pediatric CD and transmural healing in some patients. EEN was also shown to have a partial effect on severe penetrating injury associated with pediatric CD. The use of EEN treatment for severe CD has gradually increased, which may be related to the low compliance of pediatric patients with mild-to-moderate diseases (15) and a change in clinician attitudes toward the treatment.

## Exclusive enteral nutrition treatment course associated with remission

The duration of EEN treatment varied from <2 weeks to >12 weeks in different countries (16), with treatment in North America > Western Europe (17) > Japan, where the duration was only 2 weeks ± (18) (Figure 2D). Clinical symptoms usually began to resolve several days after initiating EEN, and the median time to clinical remission was 11 days to 2.5 weeks (19). Inflammatory markers were usually reduced in 1 week (19), while improvement in inflammation and nutrition took several weeks. Endoscopic and histological studies also showed that mucosal healing required about 8 weeks of EEN (9, 20). Thus, the 2020 consensus guideline of the European Crohn’s and Colitis Organization (ECC) and ESPGHAN (1) recommended 6–8 weeks of treatment for EEN-induced remission.

## Strictness of exclusive enteral nutrition implementation

Exclusive enteral nutrition is significantly better at relieving symptoms in children with active CD than PEN (6, 21) and is associated with a stronger decline in the pediatric CD activity index (PCDAI) than PEN (47% of total energy) after 6 weeks of treatment (22). More EN consumption was also associated with a higher remission rate in adults (23, 24). Thus, the stringency of execution correlates with the efficacy of EEN. ART12Q showed that EEN strictness varied by country. Some minor reforms were made to improve the compliance of children, including adding flavorings to reduce taste fatigue, creating high-energy-density formulas with small volumes, and permitting the consumption of small amounts of other beverages.

Partial enteral nutrition is not often used alone to induce remission but can supplement the induction of remission or be used in patients with mild disease and a low risk of recurrence. Sigall-Boneh et al. reported that a 50% PEN diet plus a structured exclusion diet was associated with a 70% remission rate in children with mild-to-moderate CD (25). A retrospective study by Wilschanski et al. found that consumption of a normal diet during the day and PEN at night (through continuous nasogastric tube feeding) could prolong remission and improve linear growth (26). Thus, PEN is a useful substitute for inducing remission in children with mild-to-moderate CD who cannot strictly adhere to EEN (27).

## Exclusive enteral nutrition formulation

Exclusive enteral nutrition formulations include element formula (EF; amino acid type), semi-element formula (SEF; oligopeptide type), and polymeric formula (PF; integral protein type). ART12Q found that PF, at a concentration of 1 kcal/ml, was the most used. While clinicians in Western Europe, Oceania, and Israel all prefer PF, doctors and patient families in Japan are willing to adopt EF. However, meta-analyses (28) and clinical research studies (8, 29) found no difference in the efficacy of EF, SEF, and PE in treating CD. There was also no evidence that dietary protein sources would affect treatment success. Thus, except for special cases, such as patients with a milk protein allergy, standard PF with a moderate fat content is recommended by ESPGHAN (14) due to its palatability and low cost.

## Developments in the exclusive enteral nutrition formula

The exclusive enteral nutrition formula is designed to reduce the complex interaction between diet and host immunity. However, different formulations, including low-fat, high-fat; supplementation with medium-chain triglycerides (MCT) (30), monounsaturated fatty acids (MUFA) (31); or anti-inflammatory substances [glutamine (32), transforming growth factor-β (33), and omega-3 (34)] are not found to cause significant clinical improvements. The addition of probiotics, prebiotics, and dietary fiber also requires further verification using randomized controlled trials (RCT). A specific carbohydrate diet (SCD) (35) was shown to have therapeutic value in treating IBD, but whether excessive carbohydrate levels are beneficial to children remains to be determined. While CDED (36) and CD-TREAT formulations are designed to mimic EEN by excluding certain components found in common foods (37), these are still immature protocols. For example, the low fermentable oligo-, di-, monosaccharide, and polyol (FODMAP) diet was shown to be effective against adult IBD but has not been fully studied in children (38, 39). In addition, the lactose-free diet (LFD) (40) may cause vitamin D deficiency and low calcium. The paleolithic, vegan, gluten-free, and food-specific IgG4 antibody-guided exclusion diets all had some effect on IBD (36), but no substantial progress in their development has been made. Since exclusion/restrictive diets may affect nutrition, psychology, and quality of life, ESPGHA does not recommend them for the treatment of children and adolescents with IBD, unless the potential benefits are higher than the risks. Research on novel formulations is promising, but findings will need to be verified by adequate RCT.

## Methods of exclusive enteral nutrition delivery

Adherence is the biggest issue associated with EEN, especially with the poor-tasting EF and SEF formulas. Feeding through a nasogastric tube (NG) or gastrointestinal stoma is often used to ensure adequate intake. While retrospective studies found no difference in the efficacy of EEN between oral and tube feeding (7), oral intake of <120% of the total daily calorie requirements may affect EEN effectiveness (41). Tube feeding may be more effective in adults because they are less receptive to single-taste diets than children, who still lack experience with rich flavors (7). ART12Q found that most children with IBD choose oral administration, potentially because of taste improvements in EEN formulations. Thus, ESPGHAN has recommended attempting oral administration first and then transitioning to NG feeding if oral intake remains inadequate.

## Drug combination during exclusive enteral nutrition-induced remission

Analysis results of the 12 studies showed that most gastroenterologists believe that combining drugs such as 5-ASA, 6-MP, AZA, CS, or infliximab with EEN achieves better remission and often prescribe these combinations for their patients. During glucocorticoid-induced remission, the early introduction of immunomodulators is beneficial for the maintenance of remission in patients with moderate to severe CD (42). However, the clinical benefits of early drug combinations during EEN-induced remission have not been confirmed. In addition, side effects, such as nausea, that are associated with immunomodulators may adversely affect EEN treatment.

## Evaluation of exclusive enteral nutrition efficacy

Both invasive and non-invasive methods are used to evaluate the efficacy of EEN to induce remission. Endoscopic evaluation following EEN-induced remission can help achieve mucosal healing, reduce the risk of long-term complications (1), and extend the remission period to 3 years (43). However, ART12Q found that most clinicians still use non-invasive indicators to evaluate EEN efficacy, including clinical PCDAI score, CRP, erythrocyte sedimentation rate (ESR), fecal calprotectin, nutrition score, blood cell count, biochemical indicators, and imaging. Invasive evaluations such as endoscopy and biopsy are only used in about 50% of cases. A comprehensive score combining fecal calprotectin, clinical score, and CRP is currently considered the most suitable non-invasive evaluation method for pediatric CD (1). While an evaluation of EEN induced-remission is typically recommended after 6–8 weeks, many medical centers suggest evaluating its effects after 2–3 weeks.

## Food reintroduction after exclusive enteral nutrition-induced remission

Analysis results of the 12 studies found that most medical centers gradually introduced low-fat, low-fiber, and low-allergen foods after EEN-induced remission. A retrospective study showed that the rate of recurrence and the maintenance of remission at 1 year was similar regardless of whether the food was reintroduced within 5 weeks or 3 days (44). An exclusion diet guided by food-specific antibodies appears to help maintain EEN-induced remission (45). While food intolerance was not common after the reintroduction of conventional foods, the necessity of low-allergen foods was not confirmed (46). Since most EEN formulas do not contain fiber (44), many doctors recommend a short-term low-fiber diet for the reintroduction of food to children; however, there is little evidence to support this. Given the lack of data required to form a standard plan for food reintroduction, ESPGHAN (14) recommends gradually reintroducing regular foods and reducing EEN use within 2–3 weeks. Fiber restriction is not suggested for children with IBD who have no evidence of gastrointestinal stenosis.

## Use of maintenance enteral nutrition for the maintenance of remission

Either MEN or PEN treatment is usually initiated after EEN-induced remission. MEN was developed to maintain remission, improve nutrition, and promote growth and weight gain. ART12Q found that PF was the most used MEN formulation, and almost all dietitians used dietary energy reference values to estimate pediatric energy requirements (47). Gkikas et al. reported that MEN, which accounts for 35% of the daily energy requirement, is sufficient to improve clinical remission (48). ART12Q found that 89% of nutritionists recommend MEN to fulfill 25–30% of their daily energy needs. However, the use of MEN after EEN has not been recommended as a standard protocol, especially in children without malnutrition. The optimal time for MEN treatment is also unclear. Some dietitians suggest using this therapy for as long as possible, while others suggest stopping treatment when maintenance drugs start to show effect, growth is stable and appropriate, and an ideal weight has been reached (49) (Figure 2C).

## Exclusive enteral nutrition side effects

While EEN is associated with minimal side effects, nausea, vomiting, diarrhea, abdominal distension, and abdominal discomfort can occur (2). Clinicians need to be aware of the risk of refeeding syndrome in severely malnourished children (50). In this patient population, it is necessary to gradually reduce intake of the normal diet by about 25% of the resting caloric intake needed per day and slowly increase the volume and concentration of EEN over several days until electrolyte levels are balanced (14).

## Factors influencing enteral nutrition management and patient feedback

Analysis results of the 12 studies identified many barriers to the successful implementation of EN, including EEN exclusiveness, compliance of the children and their families, health care resources, and cost-effectiveness (Figure 2E). These issues can be resolved by establishing a standardized EN program, personalizing adjustment, assuring effective doctor–patient communication, and solving social EN restrictions. Most patients and families expect dietary guidance and psychological support to become an integral part of IBD treatment (10, 41). Thus, an ideal EN management model should include education and training as well as a complete management team that includes gastroenterologists, dietitians, psychologists, nursing staff, and social workers. While insurance reimbursement to the national health system and private companies will need to be improved to reduce the burden of IBD on families.

## Conclusion

Enteral nutrition is a safe but underused treatment for children with IBD. However, there are still significant gaps in the global understanding and implementation of EN. This review evaluated recent survey studies and summarized the current status of EN treatment. The findings can be used to develop a standardized EN therapy for children with IBD.

## Author contributions

Y-MX and JL designed the research, performed the research, analyzed the data, and wrote the manuscript. MW, J-GZ, and L-LH analyzed the data. All authors contributed to the article and approved the submitted version.
